# Disulfidptosis: disulfide stress‐induced novel cell death pathway

**DOI:** 10.1002/mco2.579

**Published:** 2024-06-29

**Authors:** You Shuai, Zhonghua Ma, Peng Yuan

**Affiliations:** ^1^ Department of VIP Medical Services National Cancer Center/National Clinical Research Center for Cancer/Cancer Hospital Chinese Academy of Medical Sciences and Peking Union Medical College Beijing China; ^2^ Key Laboratory of Carcinogenesis and Translational Research (Ministry of Education) Department of Endoscopy Peking University Cancer Hospital & Institute Beijing China

## Abstract

The aberrant accumulation of intracellular disulfides in solute carrier family 7 member 11 (SLC7A11)^high^ cells under glucose starvation induces disulfidptosis. Disulfidptosis has shown potential in tumor diagnosis and treatment.

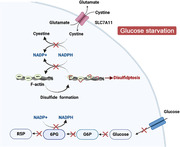

1

In a recent study published in *Nature Cell Biology*, a group led by Junjie Chen and Boyi Gan^1^ discovered a novel cell death termed disulfidptosis, identifying that glucose starvation in SLC7A11high cells induced nicotinamide adenine dinucleotide phosphate (NAPDH) depletion, aberrant disulfide bonds in actin cytoskeleton proteins and fibro actin (F‐actin) collapse, and excessive accumulation of intracellular disulfides, which led to the disulfide stress‐induced cell death. The findings reveal a nonapoptotic cell death, highlighting disulfidptosis as a prospective model for antitumor therapeutics.^1^

It is generally believed that dysregulation of intracellular disulfides, including cystine, may trigger cytotoxic effects and disulfide stress. NADPH plays a critical role in providing essential reducing power to alleviate disulfide stress, thereby safeguarding cellular viability. Cancer cells overexpressing cystine transporter SLC7A11 exhibited higher cystine uptake coupled with the depletion of NAPDH under the circumstance of glucose starvation, inducing excessive accumulation of intracellular disulfide molecules and subsequent cell death. Of note, the persistent efforts for determining novel cell death pathways have made several important new discoverie.^2^ However, the precise mechanism by which SLC7A11 dysregulation combined with glucose starvation possibly mediate a uncharacterized nonapoptotic cell death termed disulfidptosis requires to be further clarified.

In this study, Liu et al.^1^ provided the first evidence of disulfidptosis, which replied on the substantial buildup of intracellular disulfide molecules and was determined as a novel cell death distinct from all other known ones. In order to identify the mechanistic model of disulfidptosis, they developed a bio‐orthogonal chemical proteomic technique to measure alterations in the disulfide proteome induced by glucose starvation in UMRC6 cells overexpressing SLC7A11, utilizing stable isotope labeling. Results suggested that glucose starvation in cancer cells overexpressing SLC7A11 induced disulfide bonds in actin cytoskeleton proteins. Then, the authors implicated nonreducing western blots and clustered regularly interspaced short palindromic repeats (CRISPR)/CRISPR‐associated protein 9 technology to elucidate the formation of disulfide bonds in cells overexpressing SLC7A11 under the circumstance of glucose starvation. Results revealed that SLC7A11 induced cystine, when combined with glucose starvation, which was required for fostering disulfide bonding within actin cytoskeletal proteins in a fashion independent of ROS. It was elucidated that cancer cells overexpressing SLC7A11 under the circumstance of glucose starvation showed F‐actin contraction and separation from the plasma membrane. Further, authors also found that lamellipodia formation induced by Rac–WAVE regulatory complex (Rac–WRC) was involved in prompting disulfidptosis, probably owing to the branched actin network in lamellipodia offered useful markers for the disulfide bonding among actin structural proteins. In addition, the suppression of glucose transporter (GLUT) caused disulfidptosis in cancer cells overexpressing SLC7A11. Finally, Liu et al.^1^ performed a series of in vivo assays to further elucidate the mechanism of disulfidptosis. Xenograft tumor experiments demonstrated that the inhibition of GLUT obviously reduced the tumorigenicity of SLC7A11 high mice xenograft tumors. Meanwhile, the authors also introduced two lung cancer patient‐derived xenograft (PDX) models. It was suggested that GLUT inhibition effectively suppressed tumor proliferation and caused disulfide bonding within actin molecules in SLC7A11 high PDX models, contrasting with its ineffectiveness in SLC7A11 low PDX tumors. The mechanism of disulfidptosis has been depicted in Figure 1.

**FIGURE 1:**
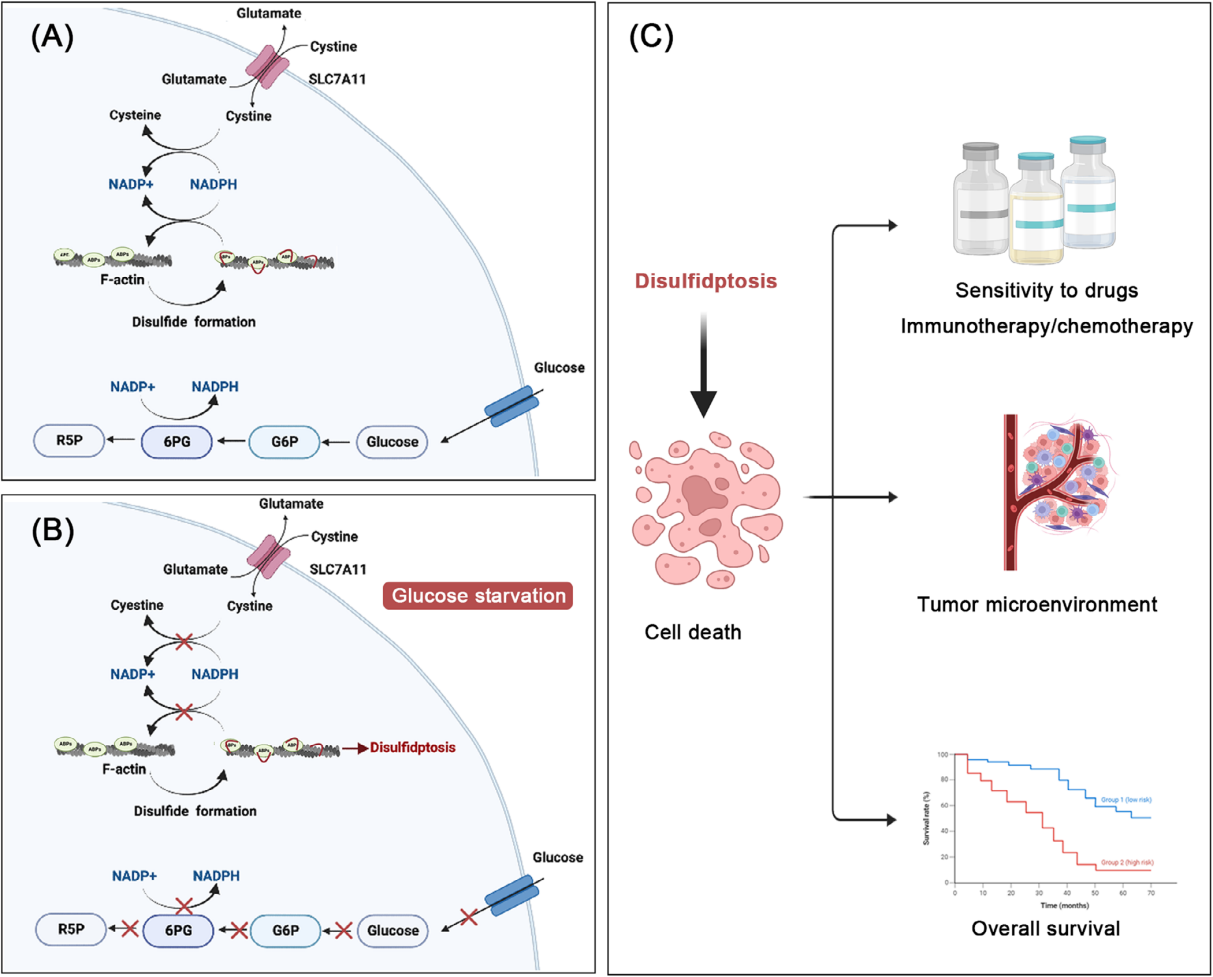
Schematic illustration of disulfidptosis mechanism. (A) In condition of high glucose, glucose enters cells and produces sufficient NADPH via the pentose phosphate pathway. Cystine is reduced by NADPH to convert it to cysteine. (B) Cancer cells overexpressing cystine transporter SLC7A11 showed high cystine uptake coupled with the depletion of NAPDH under the circumstance of glucose starvation, inducing excessive accumulation of intracellular disulfide molecules and subsequent cell death. (C) The application of disulfidptosis.

Cell death has been identified to play an essential role for organismal balance and the removal of damaged cells.^2^ Previous studies have highlighted various types of cell death, which can be categorized into regulated (RCD) and accidental (ACD) types. ACD occurs due to unexpected damage, whereas RCD is genetically regulated to maintain internal stability, covering several forms like apoptosis, necroptosis, pyroptosis, and so on. Each type of cell death is characterized by distinct triggers and molecular pathways. Apoptosis, characterized by cellular shrinkage and chromatin condensation, is activated by internal or external stimuli through caspase cascades. Importantly, the integrity of the plasma membrane is maintained throughout this process. Additionally, necroptosis represents a type of necrosis reliant on the phosphorylation of the mixed‐lineage kinase‐like protein by receptor‐interacting protein kinases 1 (RIPK1) and 3 (RIPK3). This cell death pathway is activated through stimulation of death receptors on the cell surface, as well as RNA and DNA sensing molecules within the cells. Necroptosis is marked by the permeabilization of the plasma membrane, leading to cellular and organelle swelling and the subsequent breakdown of cellular and organelle structure. Furthermore, pyroptosis is an inflammatory type of regulated cell death occurring in various cells, triggered by human caspases 1, 3, 4, 5 (mouse caspase‐11), 6, 8, and 9. Proteins from the gasdermin superfamily, essential mediators of pyroptosis, consist of a pore‐forming N‐terminal domain and a C‐terminal domain. During activation, these proteins are cleaved by inflammatory caspases, releasing the N‐terminal pore‐forming domain, which then forms oligomers and creates pores in the plasma membrane. This leads to cell swelling, chromatin degradation, and the release of inflammatory mediators, ultimately resulting in cell death. Last, ferroptosis is an iron‐dependent form of programmed cell death, marked by increased levels of cellular iron, lipid peroxides, and reactive oxygen species. This form of cell death is characterized by several unique morphological features, including a reduction in mitochondrial volume, rupture of the mitochondrial outer membrane, and diminished or absent mitochondrial cristae. Additionally, cells undergoing ferroptosis typically exhibit a nucleus of normal size without chromatin condensation, setting it apart from other cell death pathways. SLC7A11 has been observed to exert crucial function in this process, inhibiting ferroptosis by facilitating cystine import for glutathione synthesis and aiding in the detoxification of lipid peroxides. The roles of SLC7A11 in ferroptosis and disulfidptosis are both associated with redox regulation and antioxidant mechanisms. However, the functions of SLC7A11 varies due to the different triggers and molecular pathways driving these cell death processes. Of note, exploring the mechanistic model of disulfidptosis was in an urgent need. The striking study led by Junjie Chen and Boyi Gan presented novel evidence of the precise mechanism of disulfidptosis and highlighted that targeting of disulfidptosis becomes a new mode of antitumor therapy. Dysregulation of intracellular disulfides, such as cystine, mediates disulfide stress and cytotoxic effects, therefore urgent attention should be paid to strictly testifying their impact on biological activities.

In addition, in this research, the authors reported that actin polymerization and lamellipodia formation mediated by Rac–WRC prompted disulfidptosis. Thus, deep analysis are warranted to clarify additional pathways involved in mediating disulfidptosis. The understanding of disulfidptosis is nonetheless nascent in many aspects. Liu et al.^1^ mainly focused on the features of disulfidptosis in cancer cells overexpressing SLC7A11 under glucose starvation. However, the results showed that disulfidptosis can be triggered under both high and low SLC7A11 conditions, illuminating that the certain cell death was completely inhibited through preventing disulfide stress rather than other kinds of inhibitors against apoptosis, necroptosis, ferroptosis, and autophagy. Further explorations are required to identify the impact of other metabolic‐stress conditions with the depletion of intracellular NADPH level on disulfidptosis. It is worth noting that glucose starvation is rare in healthy tissues but prevalent in tumors with high metabolic demands and rapid growth, particularly in advanced‐stage patients. Thus, this research holds significant scientific and clinical translational value for patients with advanced tumors.

Recent studies have expanded the applications of disulfide death in tumors. Gan et al. demonstrated that SLC7A11 overexpression leads to NADPH depletion, causing disulfide stress and cell death under oxidative conditions like H2O2 treatment, suggesting a wider implication for this type of cell death.^3^ Further, the gene signature model across 33 cancer types has underlined the prognostic significance of disulfidptosis and its ability to improve anticancer drug sensitivity.^4^ Moreover, Zhao et al.^5^ discovered a cell cluster linked to disulfidptosis, which correlates with diminished overall survival and an aberrant tumor microenvironment. This evidence underscores the influence of disulfidptosis on precision oncology, including tumor diagnosis, prognosis, and therapeutic strategies, thereby emphasizing its clinical relevance.

The great advancements in experimental technology largely facilitate and enable the detection of disulfidptosis. The identification of disulfidptosis will be achieved through metabolic assays, morphological examinations, and protein analyses in future research. This involves assessing fluctuations in metabolites such as disulfides, NADPH, adenosine‐triphosphate（ATP, and cystine uptake, observing changes in the structure of actin filaments and tracking differences in crucial proteins like GLUT1, GLUT3, and SLC7A11. The technological advancements are of great necessity to lay a foundation for clinical implementation. However, more emphasize should be attached on the development of effective markers, which possess the characteristics of high sensitivity and specificity.

In summary, the pioneering research unlocked a previously unrecognized cell death termed disulfidptosis. The present findings of disulfidptosis not only enrich the understanding of nonapoptotic cell death but also supply tremendous impetus to develop disulfidptosis‐based cancer therapeutics through exploring the disulfide stress induced cell death. Prospectively, targeting of disulfidptosis may be a new mode of translational strategy after the ensurance of efficacy and safety for treating a variety of human cancers, especially SLC7A11 high tumors.

## AUTHOR CONTRIBUTIONS

You Shuai and Zhonghua Ma drafted the manuscript. You Shuai drew the figure. Peng Yuan conceived and finalized the manuscript. All authors have read and approved the article.

## CONFLICT OF INTEREST STATEMENT

The authors declare no conflict of interest.

## ETHICS STATEMENT

Not applicable.

## Data Availability

Not applicable.
